# Supplementary material for: Intergrated Transcriptomic and Proteomic Analysis Revealed the Differential Responses to Novel Duck Reovirus Infection in the Bursa of Fabricius of Cairna moschata
Source: Viruses. 2022 Jul 25;14(8):1615. doi: 10.3390/v14081615 (PMC9332436; doi:10.3390/v14081615)
Supplement: Supplementary file 1 [file viruses-14-01615-s001.zip › Figure S2.pdf]

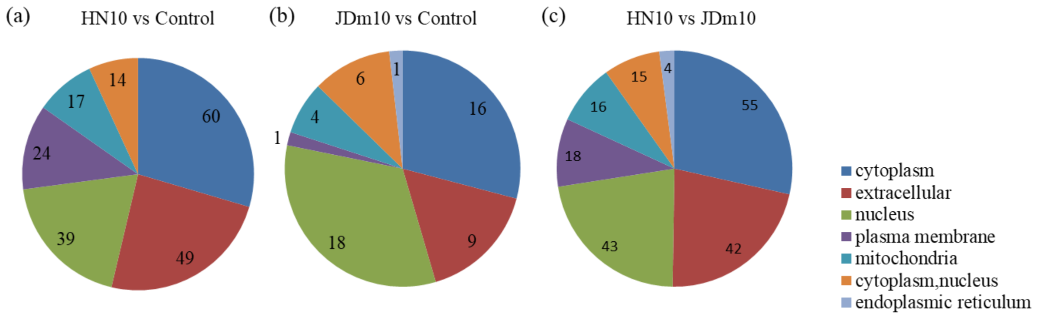

Figure S2 Subcellular locations of the DAPs in different comparisons. (a) Subcellular locations of the DAPs in the HN10 vs control comparison. (b) Subcellular locations of the DAPs in the JDm10 vs control comparison. (c) Subcellular locations of the DAPs in the HN10 vs JDm10 comparison.
