# Supplementary material for: Intergrated Transcriptomic and Proteomic Analysis Revealed the Differential Responses to Novel Duck Reovirus Infection in the Bursa of Fabricius of Cairna moschata
Source: Viruses. 2022 Jul 25;14(8):1615. doi: 10.3390/v14081615 (PMC9332436; doi:10.3390/v14081615)
Supplement: Supplementary file 1 [file viruses-14-01615-s001.zip › Figure S4.pdf]

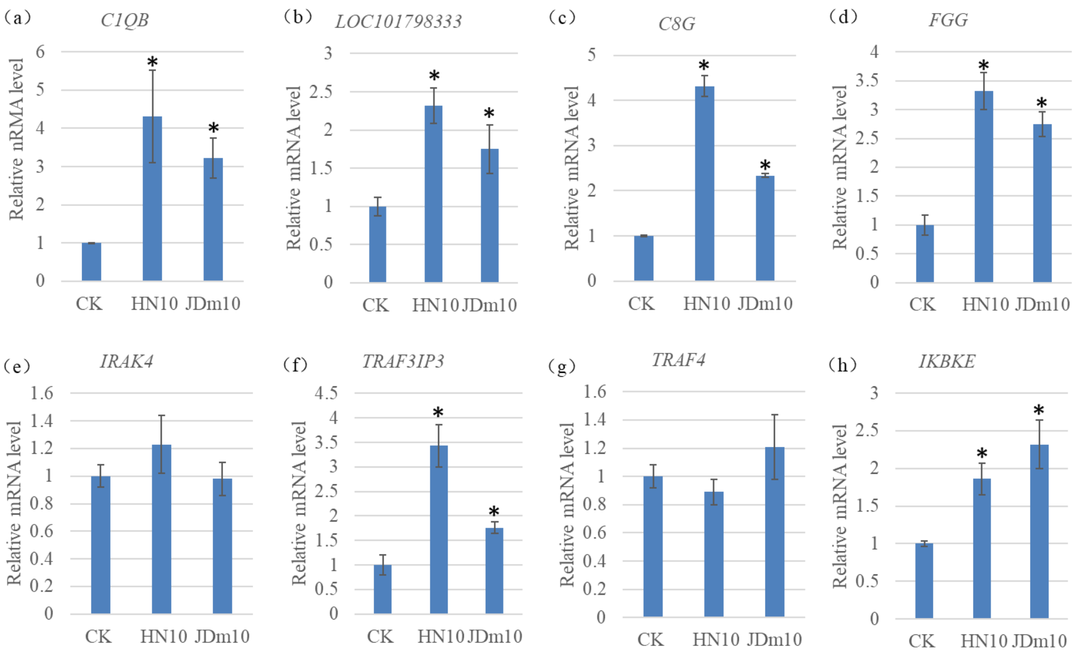

Figure S4 **QRT-PCR analysis.** The expression levels of eight key genes, including (a) C1QB, (b) LOC10179833, (c) C8G, (d) FGG, (e) IRAK4, (f) TRAF3IP3, (g) TRAF4, and (h) IKBKE were selected randomly and confirmed using qRT-PCR. "*" indicated significant differences between treatment group and CK.
