# Supplementary figures and images for: Intergrated Transcriptomic and Proteomic Analysis Revealed the Differential Responses to Novel Duck Reovirus Infection in the Bursa of Fabricius of Cairna moschata
Source: Viruses. 2022 Jul 25;14(8):1615. doi: 10.3390/v14081615 (PMC9332436; doi:10.3390/v14081615)

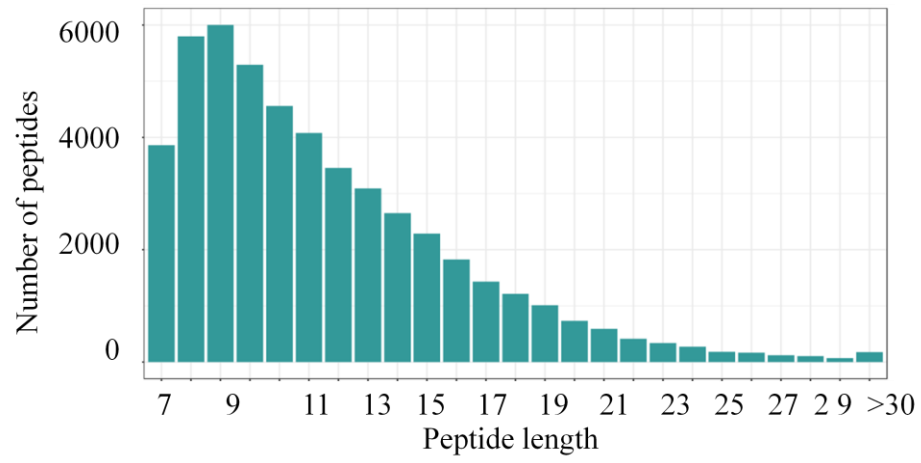

Figure S1 The peptide length of all the identified peptides.

Supplement: Supplementary file 1 [file viruses-14-01615-s001.zip › Figure S1.pdf]
