# Supplementary material for: Intergrated Transcriptomic and Proteomic Analysis Revealed the Differential Responses to Novel Duck Reovirus Infection in the Bursa of Fabricius of Cairna moschata
Source: Viruses. 2022 Jul 25;14(8):1615. doi: 10.3390/v14081615 (PMC9332436; doi:10.3390/v14081615)
Supplement: Supplementary file 1 [file Figure S3.pdf › Figure S3.pdf]

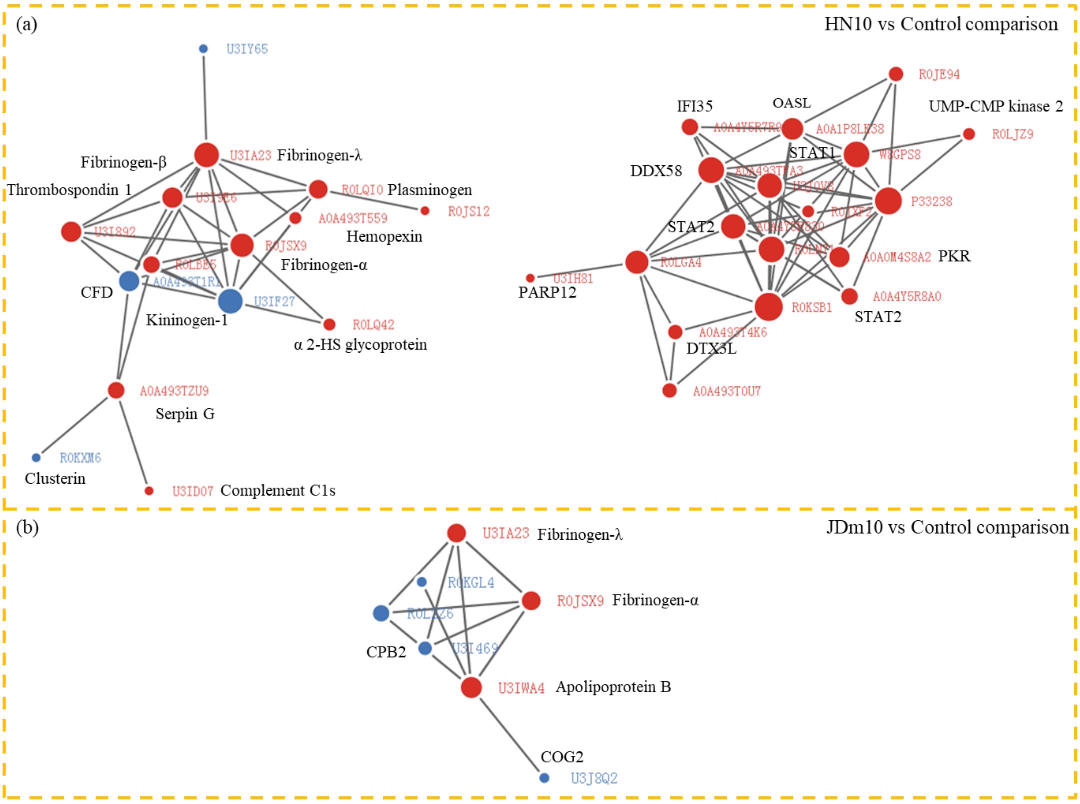

Figure S3 Protein-protein interaction (PPI) network analysis of the DAPs. (a) The PPI network for the DAPs in the HN10/control comparison. (b) The PPI network for the DEPs in the JDm10/control comparison. Cystoscope software version 3.6.1 (http://cytoscape.org/) was used for the PPI analysis.
